# Mutations in the non-coding *RNU4ATAC* gene affect the homeostasis and function of the Integrator complex

**DOI:** 10.1093/nar/gkac1182

**Published:** 2022-12-20

**Authors:** Fatimat Almentina Ramos Shidi, Audric Cologne, Marion Delous, Alicia Besson, Audrey Putoux, Anne-Louise Leutenegger, Vincent Lacroix, Patrick Edery, Sylvie Mazoyer, Rémy Bordonné

**Affiliations:** Institut de Génétique Moléculaire de Montpellier, University of Montpellier, CNRS UMR5535, 34293 Montpellier, France; INRIA Erable, CNRS LBBE UMR 5558, University Lyon 1, University of Lyon, 69622 Villeurbanne, France; Université Claude Bernard Lyon 1, INSERM, CNRS, Centre de Recherche en Neurosciences de Lyon U1028 UMR5292, GENDEV, 69500 Bron, France; Université Claude Bernard Lyon 1, INSERM, CNRS, Centre de Recherche en Neurosciences de Lyon U1028 UMR5292, GENDEV, 69500 Bron, France; Université Claude Bernard Lyon 1, INSERM, CNRS, Centre de Recherche en Neurosciences de Lyon U1028 UMR5292, GENDEV, 69500 Bron, France; Clinical Genetics Unit, Department of Genetics, Centre de Référence Anomalies du Développement et Syndromes Polymalformatifs, Hospices Civils de Lyon, University Lyon 1, Bron, France; Inserm, Université Paris Cité, NeuroDiderot, UMR1141, 75019 Paris, France; INRIA Erable, CNRS LBBE UMR 5558, University Lyon 1, University of Lyon, 69622 Villeurbanne, France; Université Claude Bernard Lyon 1, INSERM, CNRS, Centre de Recherche en Neurosciences de Lyon U1028 UMR5292, GENDEV, 69500 Bron, France; Clinical Genetics Unit, Department of Genetics, Centre de Référence Anomalies du Développement et Syndromes Polymalformatifs, Hospices Civils de Lyon, University Lyon 1, Bron, France; Université Claude Bernard Lyon 1, INSERM, CNRS, Centre de Recherche en Neurosciences de Lyon U1028 UMR5292, GENDEV, 69500 Bron, France; Institut de Génétique Moléculaire de Montpellier, University of Montpellier, CNRS UMR5535, 34293 Montpellier, France

## Abstract

Various genetic diseases associated with microcephaly and developmental defects are due to pathogenic variants in the U4atac small nuclear RNA (snRNA), a component of the minor spliceosome essential for the removal of U12-type introns from eukaryotic mRNAs. While it has been shown that a few *RNU4ATAC* mutations result in impaired binding of essential protein components, the molecular defects of the vast majority of variants are still unknown. Here, we used lymphoblastoid cells derived from *RNU4ATAC* compound heterozygous (g.108_126del;g.111G>A) twin patients with MOPD1 phenotypes to analyze the molecular consequences of the mutations on small nuclear ribonucleoproteins (snRNPs) formation and on splicing. We found that the U4atac108_126del mutant is unstable and that the U4atac111G>A mutant as well as the minor di- and tri-snRNPs are present at reduced levels. Our results also reveal the existence of 3’-extended snRNA transcripts in patients’ cells. Moreover, we show that the mutant cells have alterations in splicing of *INTS7* and *INTS10* minor introns, contain lower levels of the INTS7 and INTS10 proteins and display changes in the assembly of Integrator subunits. Altogether, our results show that compound heterozygous g.108_126del;g.111G>A mutations induce splicing defects and affect the homeostasis and function of the Integrator complex.

## INTRODUCTION

Two different splicing machineries exist in eukaryotes: the major or U2-dependent spliceosome excises the vast majority of introns, whereas the U12-dependent spliceosome removes ∼850 U12-type introns located in ∼750 genes (1–5). The U2-dependent spliceosome contains U1, U2, U4 and U6 small nuclear RNAs (snRNAs) and the U12-dependent machinery contains the functional counterparts U11, U12, U4atac and U6atac, the U5 snRNA being common to both spliceosomes. These snRNAs associate with proteins to form small nuclear ribonucleoproteins (snRNPs), which are the building blocks of the spliceosomes. Among the snRNPs, the U4/U6.U5 tri-snRNP is the largest pre-assembled spliceosomal complex, containing >30 proteins in addition to U5 snRNA and base-paired U4/U6 snRNAs (1,2,6). Immunoprecipitation studies indicate that the minor U4atac/U6atac/U5 snRNP contains most proteins associated with the major tri-snRNP, namely the U5-specific 220, 116, 100 and 40 kDa proteins and the tri-snRNP-specific 110, 65 and 27 kDa proteins (7). The minor tri-snRNP also contains the U4/U6-specific 60 and 61 kDa proteins, the latter being essential for both minor and major tri-snRNP formation (7). The amount of the minor snRNPs is ∼1/100 the level of the major snRNPs (8,9).

The biogenesis of minor and major snRNPs proceeds through similar pathways and is an ordered multistep process (6,10). With the exception of U6 and U6atac which are transcribed by RNA polymerase (RNApol) III, the major and minor U-snRNAs are synthesized by RNApol II in the nucleus as immature precursors that contain a m^7^G cap structure and extra nucleotides at the 3′ end. The formation of the 3′ end of the human snRNAs relies on the Integrator, a multisubunit complex promoting endonucleolytic cleavage of the nascent snRNAs (11,12). The snRNA transcripts are cleaved upstream of the 3′ box which is a conserved but degenerate sequence that is located 9–19 nt downstream of the mature 3′ end of the snRNA (13). The association of the common Sm core proteins with the Sm site of the snRNA occurs in the cytoplasm and is regulated by the survival motor neuron (SMN) complex and protein arginine methyltransferases (14–17). The binding of the Sm core to the snRNA is required for the hypermethylation of the m^7^G cap structure to an m_3_G cap by the Tgs1 (trimethylguanosine synthase) hypermethylase (18–20) and for the 3′ end trimming of the extra nucleotides by the TOE1 deadenylase (21,22).

Mutations in the *RNU4ATAC* gene are responsible for the autosomal recessive disorder named microcephalic osteodysplastic primordial dwarfism type 1 (MOPD1, OMIM 210710) (23–25). It is a very rare (<1 in 1 000 000 live births) and severe disorder which is characterized by dwarfism, intellectual disability and multiple malformations including severe microcephaly and cortical brain malformations, severe ante- and postnatal growth retardation, dysmorphic features and ocular/auditory sensory defects. Early unexplained death occurred within the first 2 years of life in >70% of the published cases (25). Other rare congenital disorders, with less severe phenotypes and named Roifman syndrome (RFMN, OMIM 616651) and Lowry Wood syndrome (LWS, OMIM 226960), have also been assigned to biallelic *RNU4ATAC* mutations (26,27). Both RFMN and LWS have features overlapping with MOPD1 (i.e. microcephaly, growth retardation, skeletal dysplasia and intellectual disability), but these disorders are not associated with early mortality, they do not include visible structural brain anomalies and they have less pronounced microcephaly and growth retardation.

Most MOPD1 mutations are located in the 5′ stem–loop of U4atac (25), while those associated with RFMN, most of them of the compound heterozygous type, appear to be located in the stem II domain of U4atac for at least one of the two mutations (26,28). Concerning the variants responsible for LWS, they are found in the different functional regions of U4atac including the stem II, the 5′ stem–loop, the 3′ stem–loop and the Sm-binding site (27,29). While it was shown that the recurrent g.51G>A MOPD1 mutation gives rise to decreased binding of the spliceosomal 15.5K and PRPF31 proteins and leads to decreased assembly of minor di-snRNP and/or tri-snRNP particles (30), the mechanisms responsible for the impairment of minor splicing in the case of other U4atac variants are still unknown. In this study, we determined the molecular defects occurring in cells of new dizygotic twin patients showing MOPD1 phenotypes and found to be compound heterozygotes combining the g.108_126del deletion with the g.111G>A mutation in the 3′ end of the *RNU4ATAC* gene. Using patients’ lymphoblastoid cells, we found that the U4atac108_126del snRNA is unstable and that the levels of the U4atac111G>A snRNA and minor di- and tri-snRNPs are slightly lower than expected. By transcriptomic profiling, we also found that heterozygous mutant cells contain 3′-extended snRNA species and exhibit alterations in splicing of minor introns. Finally, we show that the levels of the INTS7 and INTS10 Integrator proteins, encoded by genes carrying minor introns, are reduced in patients and that formation of large macromolecular Integrator complexes is impaired in mutant cells.

## MATERIALS AND METHODS

### Patients, *RNU4ATAC* mutation detection and control

Three-month-old twin sisters were referred to the Clinical Genetics Unit of the Hospices Civils Hospital (Lyon, France) and, based on their clinical phenotype suggestive of MOPD1, were screened for mutation in *RNU4ATAC* after written informed parental consent was obtained (Gauthier et al., in preparation). Genomic DNA was extracted from a peripheral blood sample from both sisters and both of their parents. *RNU4ATAC* analysis was performed by Sanger sequencing. The amplicon, including the non-coding RNA sequence (NR_023343.1) and 100 surrounding nucleotides, was amplified by polymerase chain reaction (PCR; Forward primer: TAGGGCGAGGCTCACGAATT, Reverse primer: AGACTACTGGGCTGACTCAG) and sequenced on an ABI 3730xl DNA Analyzer or an ABI 3130xl DNA Analyzer with BigDye® Terminator v3.1 (Applied Biosystems). At the age of 1 year, a new peripheral blood sample was taken and used to establish a lymphoblastoid cell line by Epstein–Barr virus (EBV) transformation following standard procedures, by the Lyon University Hospital Biobank dedicated to genetic diseases for processing, storage and management (CBC Biotec of the Hospices Civils de Lyon, certified with a specific French standard for biobanks, NF S96-900), after informed written consent for the use of these samples in research was obtained from the parents. The C702 control lymphoblastoid cell line, also established and provided by the CBC Biotech, originated from a peripheral blood sample taken from a 2-month-old girl whose parents signed an informed consent for use in research.

### Cell culture and extract preparation

Lymphoblastoid cell cultures were performed in recommended medium (RPMI 1640, 2 mM l-glutamine, 10% fetal bovine serum) in tissue culture flasks at 37°C under 5% carbon dioxide. Whole-cell extracts were prepared from harvested cells in HNTG buffer (20 mM HEPES, pH 7.9, 150 mM NaCl, 1% Triton X-100, 10% glycerol, 1 mM MgCl_2_, 1 mM EGTA, 1 mM phenylmethylsulfonyl fluoride, protease inhibitor mixture). After lysis on ice for 20 min, lysates were centrifuged at 15 000 *g* (10 min, 4°C) and supernatants were carefully removed and used for western analysis, immunoprecipitation experiments and glycerol gradient sedimentation.

### Glycerol gradient sedimentation analysis

Extracts were diluted 3-fold with buffer A (50 mM Tris–HCl pH 7.4, 150 mM NaCl, 5 mM MgCl_2_) and layered on 11 ml of 10–30% (w/v) glycerol gradients in the same buffer. Centrifugation was performed at 37 000 rpm for 14 h in an SW41 rotor at 4°C. Fractions of 500 μl were recovered and extracted with an equal volume of phenol–chloroform, ethanol precipitated and resuspended in 10 μl of loading buffer (99% formamide, 0.02% xylene cyanol, 0.02% bromophenol blue). The RNA samples were then subjected to northern blot analysis. For analyses of Integrator subunits, fractions of 250 μl were mixed with 1 ml of cold acetone and incubated at –20°C for 2 h. After centrifugation at 13 000 *g* for 15 min, the supernatant was carefully removed and acetone was allowed to evaporate at room temperature for 30 min. The pellet was dissolved in sodium dodecylsulfate (SDS) sample buffer and analyzed on a Mini-Protean 4–15% TGX gel (Biorad).

### Northern blot analysis

Total RNA was purified from cells with Tri-Reagent (Sigma) according to the manufacturer's procedure. RNA samples were separated on a 6% TBE–urea gel (Invitrogen) and transferred electrophoretically to a Nytran membrane in 1× TBE buffer at 35 V for 2 h at room temperature. After UV treatment, the membrane was pre-hybridized for 1 h in 6× SSC, 10× Denhart's solution and 0.2% SDS at 65°C. Hybridization was performed overnight at 25°C in 6× SSC, 5× Denhart's solution and 0.2% SDS with ^33^P-5'-end-labeled oligonucleotide. Filters were washed twice for 15 min at 32°C in 6× SSC and 0.2% SDS, exposed on a Storage Phosphor screen and analyzed using the Typhon 9200 scanner and ImageQuant Software. For more stringent washing conditions, blots were washed three times for 30 min at 52°C. The sequences of the oligonucleotides used as probes are shown in Supplementary Table S1.

### Immunoprecipitation experiments

Antibodies were pre-coupled with 30 μl of protein A–Sepharose CL-4B beads for 2 h at 4°C. After three washes with IP150 buffer (20 mM Tris–HCl pH 7.4; 150 mM NaCl, 5 mM MgCl_2_, 0.1% Nonidet P-40), extracts were added and rotated with beads for 2 h at 4°C. To analyze RNA, after five washes with IP150 buffer, the immunopellet was extracted with an equal volume of phenol–chloroform, ethanol precipitated and analyzed by northern blot. For protein analyses, the immunopellet was resuspended in 1× Laemmli buffer and analyzed by SDS–polyacrylamide gel elctrophoresis (PAGE) and western blot. The antibodies used in this work are described in Supplementary Table S1.

### RNA 3′ end determination using rapid amplification of cDNA ends (RACE)

Determination of the 3′ end of U4atac processing intermediates was performed using the RACE system according to the manufacturer's procedure (Cat. no. 03353621001, Roche). Total RNA (2 μg) was used for cDNA amplification in a final volume of 20 μl with 1 μl of oligo(dT) anchor primer (37.5 μM) and 10 U of transcriptor reverse transcriptase. The reaction was incubated for 1 h at 55°C followed by an additional 5 min incubation at 85°C. The tube was shifted to ice and 10 μl of water was added to the reaction. RACE was followed by PCR amplification using the forward U4atacF3 primer and the reverse anchor primer. A 1 μl aliquot of the above reverse transcription reaction was used in a 50 μl PCR with 1 U of Pfu DNA polymerase (Promega) and 1 μl of each primer (12.5 μM). The 3′ RACE PCR products were analyzed on a 2% agarose gel to visualize extended U4atac transcripts. Agarose bands containing U4atac processing intermediates and U4atac extended products were subjected to Nucleospin Gel and PCR purification columns (Macherey-Nagel), and directly cloned into the pIIIMS2-2 vector. After transformation into *Escherichia coli*, individual clones were selected and sequenced using adequate primers. Oligonucleotides used for 3′ RACE and reverse transcription–PCR (RT–PCR) validation experiments are listed in Supplementary Table S1.

### RNA-seq library preparation and analysis

RNA sequencing experiments were performed by Integragen Genomics (Evry, France). Libraries were prepared with the NEBNext UltraII Directional RNA Library Prep Kit for the Illumina protocol according to the supplier’s recommendations with the purification of poly(A)^+^ RNA using poly(T) oligo-attached magnetic beads from 100 ng of total RNA treated with RQ1 RNase-free DNase (Promega). A fragmentation using divalent cations under elevated temperature was used to obtain ∼300 nt pieces, followed by double-stranded cDNA synthesis and finally Illumina adapter ligation and cDNA library amplification by PCR for sequencing.

The RNA-seq experiments were performed on a NovaSeq6000 sequencer (Illumina), yielding ∼35 million stranded paired-end reads of 100 bp. Image analysis and base calling were performed using Illumina Real Time Analysis (3.4.4) with default parameters.

The U12 introns were identified using the GRCh38 version of the genome, the Ensembl95 version of the annotation and T. Alioto script used for the U12DB (http://genome.crg.es/cgi-bin/u12db/u12db.cgi) (31). A total of 869 U12 introns in 718 genes were identified. Differential expression analysis was done with RSEM v1.3.1 (32) for gene quantification and DESeq2 v1.32.0 (33) for the differential analysis. IRFinder v1.2.5 (34) was used for PSI quantification and kissDE v1.15.0 (35) was used for the differential splicing analysis.

Analyses of the coverage of the 3′ region of snRNAs carrying an Sm site was performed by retrieving the human snRNAs from Ensembl BioMart. For each snRNA in the annotation, we examined the BAM alignment files corresponding to the control and patient AC438/AC439 lymphoblastoid cells and, for each alignment file, we extracted the number of reads aligning in the snRNA and the 3′ region of the snRNA (100 bases after the last 3′ annotated base of the snRNA) with pysam.

### Western blot analysis

For western blot analysis, the protein content of the fractions was determined by using the BCA protein assay kit (Pierce), and equal amounts of proteins from each lysate were analyzed. Samples were boiled in SDS sample buffer and analyzed on a Mini-Protean 4–15% TGX gel (Biorad). Proteins were blotted on a Protran nitrocellulose membrane (Amersham) using standard procedures and incubated with antibodies followed by either anti-rabbit or anti-mouse secondary antibodies. Detection was carried out by enhanced chemiluminescence (Pierce). Imaging and quantification of chemiluminescent signals were performed using the Fiji imaging system. Antibodies used for western blot analysis are listed in Supplementary Table S1.

### Statistical analysis

GraphPad Prism v.5.0 was used for statistical analysis. The number of replicates and the type of tests are indicated in the figure legends. Statistical significance is indicated by asterisks in the figures with *P ≤ 0.05, **P ≤ 0.01 and ***P ≤ 0.001.

## RESULTS

### Characterization of compound heterozygous g.108_126del;g.111G>A mutations

**Figure 1.**
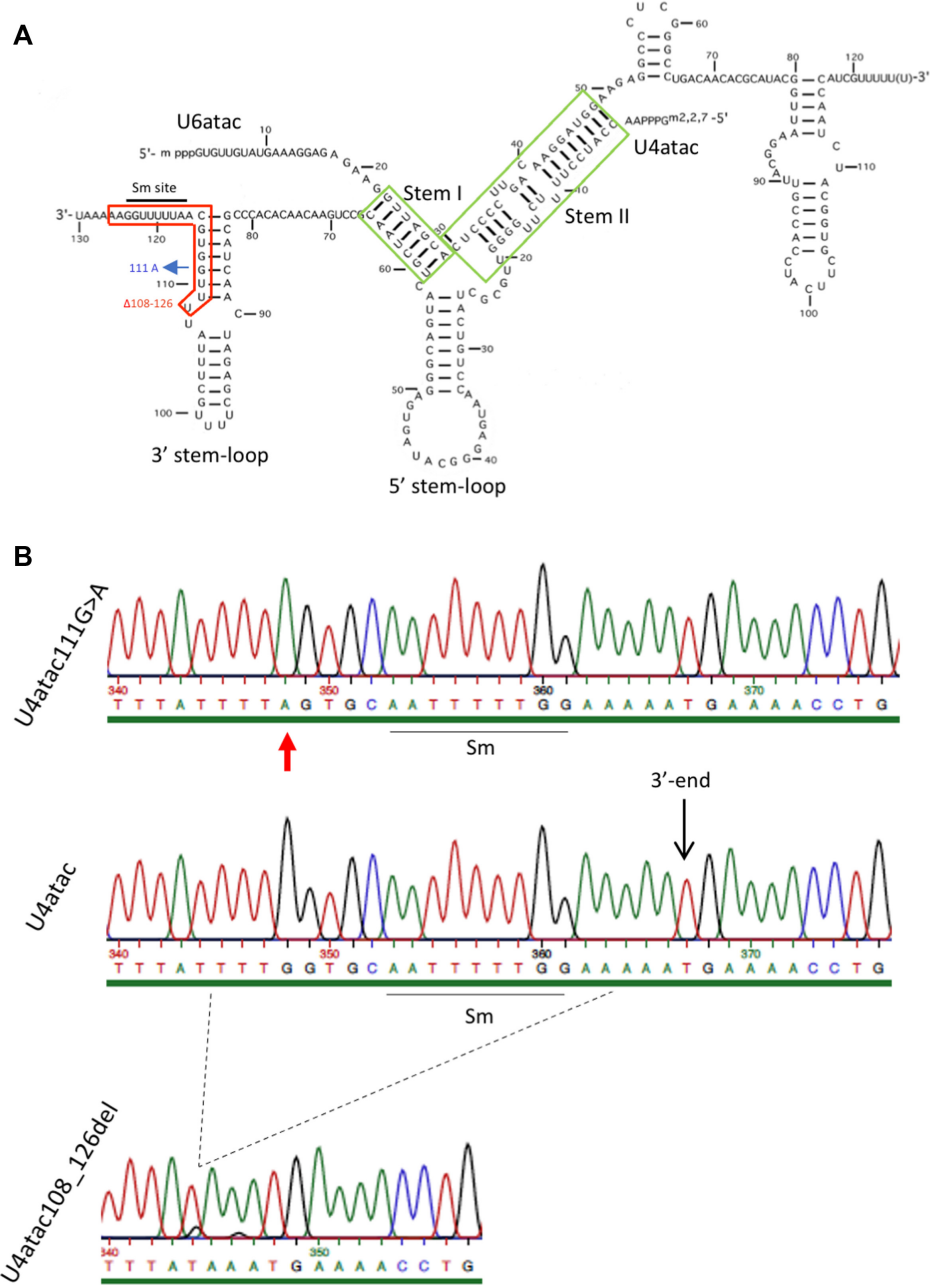
(**A**) Secondary structure of the human U4atac/U6atac snRNAs. The mutations found in the compound heterozygous g.108_126del;g.111G>A *RNU4ATAC* twin sisters are shown in red and blue. Adapted from Padgett and Shukla (91). (**B**) Sequencing chromatograms showing mutations in cloned DNA extracted from immortalized lymphoblastoid cells derived from compound heterozygous *RNU4ATAC* patients. The corresponding wild-type sequence from the control is shown in the middle. The Sm site essential for Sm core complex binding and snRNA stability is underlined. The 3′ end of the wild-type U4atac is indicated. The position of the 111G>A mutation in the mutant is shown by a red arrow and the 108_126 deletion by dashed lines.

Infant twin sisters with hallmark features of MOPD1 were found to be compound heterozygotes in the *RNU4ATAC* gene for g.108_126del;g.111G>A mutations (Gauthier et al., in preparation). The 108_126del deletion of 19 nt encompasses part of the apical stem of the intramolecular 3′ stem–loop and the entire Sm-binding site, while the 111G>A mutation disrupts a base pairing in the apical stem of the 3′ stem–loop (Figure 1A). We first confirmed the presence of these mutations in EBV-immortalized lymphocytes isolated from both patients, named hereafter AC438 and AC439. Genomic DNA was extracted from patients’ cells as well as from a control lymphoblastoid cell line (C702) and used for PCR amplification of the *RNU4ATAC* locus with specific primers. PCR products were subcloned and the sequences of individual clones were determined using classical Sanger sequencing. As shown in Figure 1B, both the 108_126del and the 111G>A mutations can be seen in the *RNU4ATAC* gene in immortalized lymphocytes of patients AC438 and AC439, but not in control cells.

### Expression of mutant U4atac snRNAs in lymphoblastoid cells derived from patients AC438 and AC439

**Figure 2.**
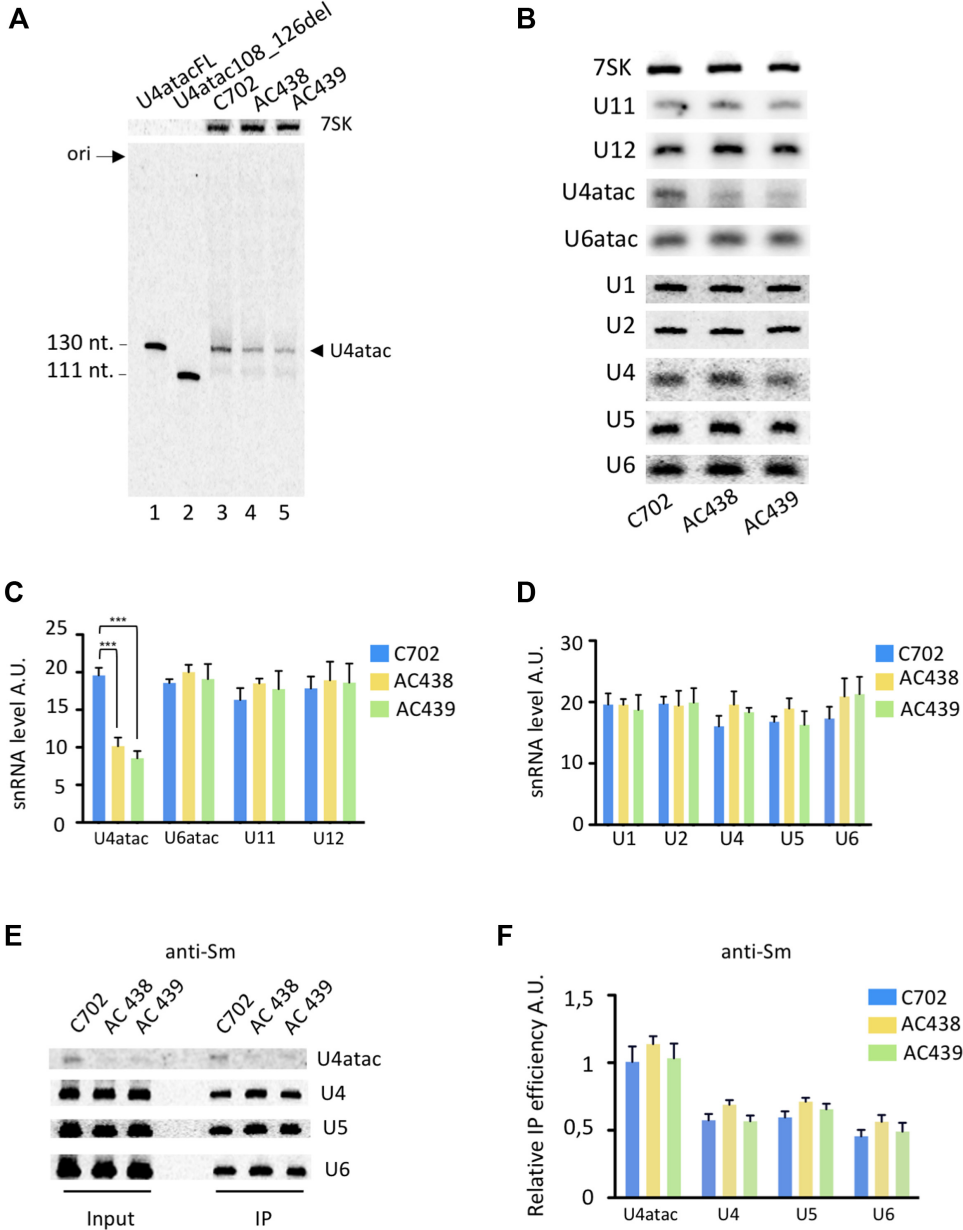
Expression of snRNAs. (**A**) Northern blot analysis of U4atac from cells of control (C702) (lane 3) and patients AC438 (lane 4) and AC439 (lane 5) . A probe complementary to nucleotides 67–87 of U4atac was used. Electrophoresis of total RNAs was performed together with 0.1 ng of DNA oligonucleotides of sizes and sequences corresponding to full-length U4atac (130 nt) (lane 1) and to the U4atac108_126del (111 nt) (lane 2) species. Washing of the blot was performed under stringent conditions (52°C). The 7SK RNA was used as a loading control. (**B**) Northern blot analysis of minor and major snRNAs found in control and patients’ lymphoblastoid cells. The 7SK RNA served as loading control and was used for quantification. (**C, D**) Quantification was performed from three trials. Data are presented as bar graphs with standard deviation (SD) and ***(*P* < 0.001) represents statistical significance as determined by one-way analysis of variance (ANOVA) with Tukey's test. A.U., arbitrary units. (**E, F**) Extracts from control C702 and AC438/AC439 patients’ lymphoblastoid cells were used in immunoprecipitation experiments with anti-Sm antibodies. The input and the immunoprecipitated (IP) RNAs were analyzed by northern blot and probed with radiolabeled oligonucleotides complementary to the indicated snRNAs. Quantification (F) was performed from two trials. The relative IP efficiency represents the ratio of band intensities found in the IP pellet compared with the input; A.U., arbitrary units. Error bars represent the SD. No significant differences in the immunoprecipitation efficiency were found.

As a first step in characterizing the defects occurring in cells expressing the U4atac mutants, we performed northern blot analyses on total RNA isolated from control and patients’ lymphoblastoid cells using DNA oligonucleotides carrying the sequences of wild-type U4atac (130 nt) and mutant U4atac108_126del (111 nt) transcripts. As shown in Figure 2A, a band corresponding to the U4atac111G>A snRNA is detected and found at ∼55% lower levels in the mutant cells when compared with the amount of U4atac snRNA found in the control. In contrast, no band corresponding to the U4atac108_126del mutant snRNA (111 nt) can be detected, suggesting that this deletion mutant lacking the Sm site is unstable. We next tested the levels of the other minor and major spliceosomal snRNAs as well as the 7SK snRNA (used as loading control) and found that their steady-state levels are approximately similar in the C702 control and in both AC438 and AC439 patients’ cells (Figure 2B–D).

### The U4atac111G>A mutant binds to Sm proteins

During snRNP synthesis, the association of the heptameric ring of Sm proteins with the Sm site of the snRNA is a prerequisite for the maturation, stability and function of the snRNPs (36). To determine whether the U4atac111G>A is able to bind to Sm proteins, we performed immunoprecipitation experiments with anti-Sm antibodies followed by northern blot analyses. As shown in Figure 2E and consistent with the fact that only U4atac111G>A can be detected in these cells, lower levels of U4atac snRNAs are found in the pellet in patients' cells compared with the wild type, while similar amounts of spliceosomal U4, U5 and U6 snRNAs are immunoprecipitated. However, the immunoprecipitation efficiency is equivalent in control and mutant cells (Figure 2F), demonstrating that the U4atac111G>A snRNA retains the ability to associate with the Sm core protein complex.

To evaluate further the formation of the U4atac111G>A-containing snRNPs, we performed immunoprecipitation experiments using antibodies against U5-100K (PRPF28) and U5-40K, which are components of the U5 snRNP and associate with the minor and major tri-snRNPs (37,38). As shown in Supplementary Figure S1, only background levels of U4atac and U4atac111G>A are found in the pellet from control and patients’ extracts. The failure to immunoprecipitate U4atac and U4atac111G>A with anti-100K and anti-40K could be due to the low titer/affinity of these antibodies and to the low levels of the minor snRNPs (1/100 the level of the major snRNPs) in both control and mutant cells.

### Examination of snRNP profiles by glycerol gradient sedimentation

**Figure 3.**
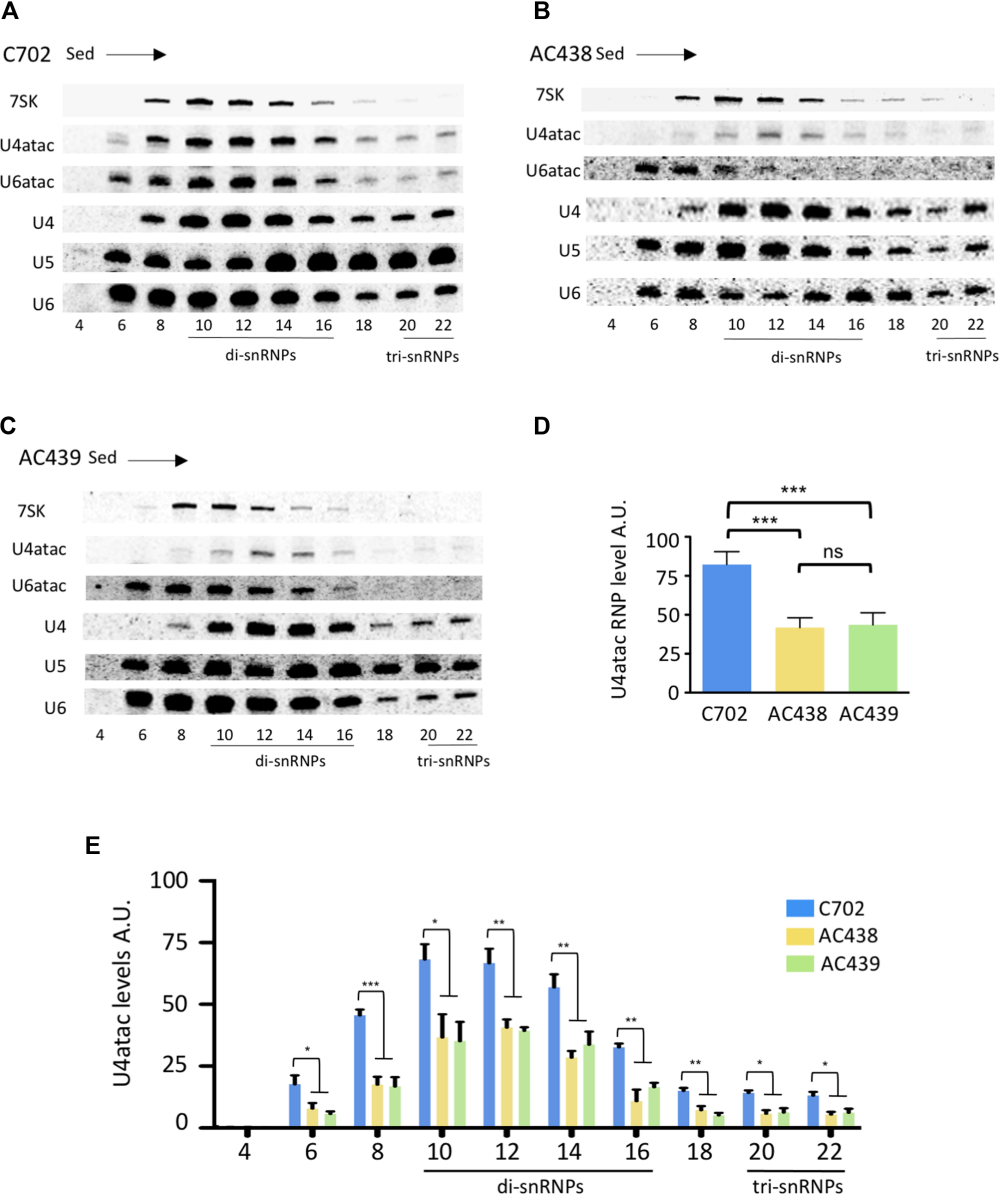
Analyses of snRNPs profiles. (**A–C**) Glycerol gradient sedimentation of major and minor snRNPs. Extracts prepared from the control (C702) and AC438/439 patients’ cells were fractionated and RNAs present in each fraction were analyzed by northern blot. The blots were probed with 5′-labeled [^33^P]oligonucleotides complementary to the indicated snRNAs. The fraction numbers and the positions of snRNP complexes are shown. The direction of sedimentation, indicated by the arrow, is from top to bottom. (**D**) Quantification of U4atac levels in control and AC438/439 patients’ cells was performed from three trials by using the 7SK RNA as loading control. A.U., arbitrary units. Data are presented as bar graphs with the SD. ns (non-significant), ***(*P* < 0.001) represent statistical significance as determined by one-way ANOVA with Tukey's post-hoc test. (**E**) Levels of minor di- and tri-snRNPs were determined by quantification of U4atac in control and AC438/439 patients’ cells. Data are presented as bar graphs with the SD. *(*P* < 0.05), **(*P* < 0.01) and ***(*P* < 0.001) represent statistical significance as determined by one-way ANOVA with Tukey's post-hoc test. A.U., arbitrary units.

We next examined the profile of snRNPs in extracts prepared from control or patients’ lymphoblastoid cells by performing glycerol gradient sedimentation followed by northern blot analysis. As shown in Figure 3A, a clear separation of U4atac snRNPs is observed in control C702 cells, with U4atac being found as di-snRNPs in fractions 10–16 and as tri-snRNPs in fractions 20–22 at the bottom of the gradient. The U4atac111G>A snRNA is also found in fractions 10–16 and in fractions 20–22 in the AC438 (Figure 3B) and AC439 (Figure 3C) patients’ cells, with a steady-state reduction of ∼55% compared with control as determined by quantification (Figure 3D). While the amounts of the major U4, U5 and U6 spliceosomal snRNAs are equivalent in control and mutant cells in the different fractions, the level of the U6atac snRNA is lower in fractions corresponding to the minor di- and tri-snRNPs in the mutant cells. Quantification of the minor U4atac/U6atac and U4atac/U6atac/U5 particles indicates a decrease of ∼55% in the AC438 and AC439 patients’ cells compared with the control (Figure 3E). Altogether, these results show that patients’ cells contain less U4atac and U6atac snRNAs in the di- and tri-snRNP fractions.

### The AC438 and AC439 patients’ cells contain 3′-extended spliceosomal snRNA species

**Figure 4.**
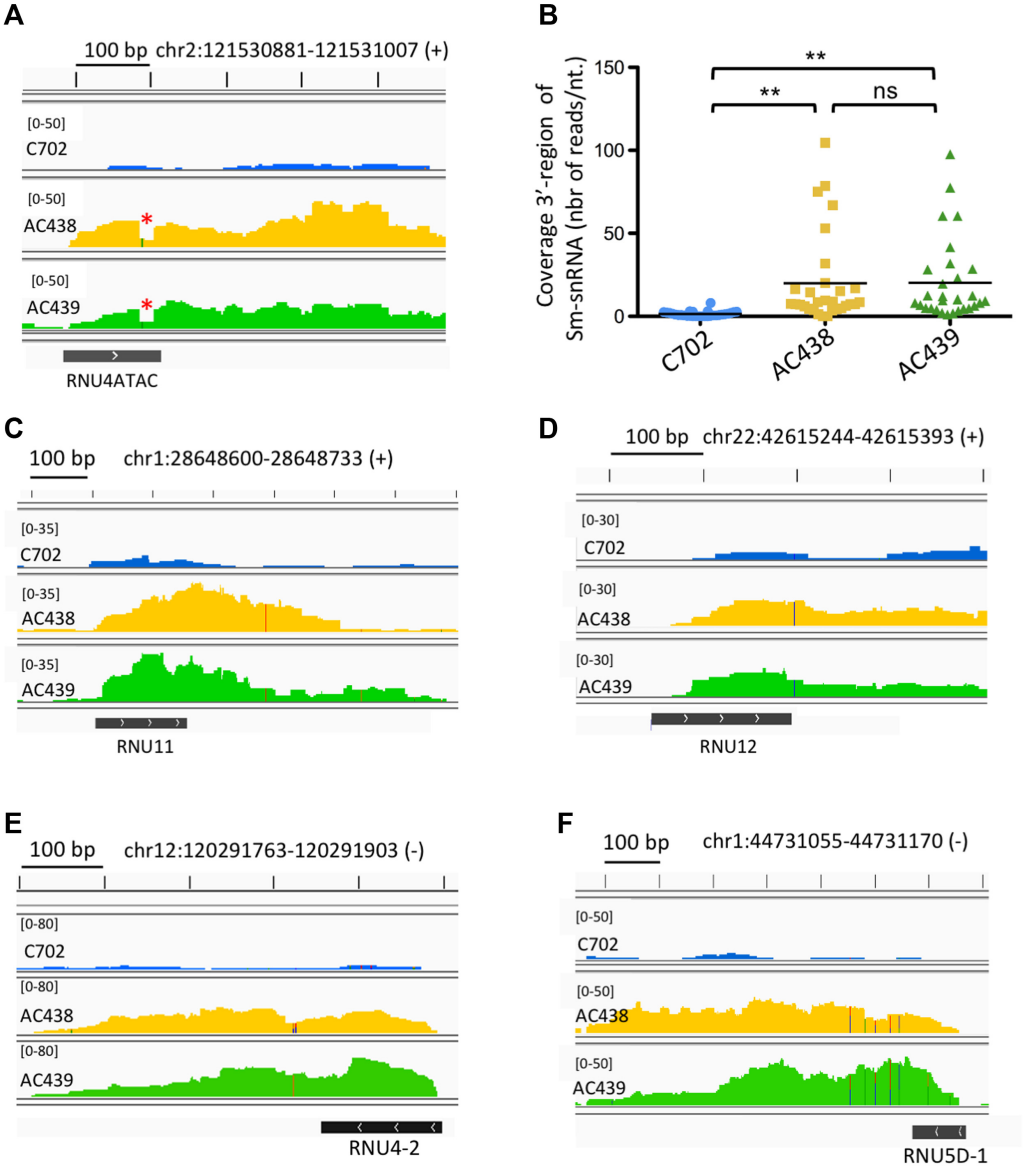
Genome-wide analysis of 3′-extended snRNAs. (**A**) Genomic read coverage observed in the U4atac region in the RNA-seq experiment performed on poly(A)^+^ RNA purified from control (C702) and AC438/AC439 patients’ cells. The drop in reads covering the 108_126del region is indicated by a red asterisk. The chromosomal location of *RNU4ATAC* is shown and the read coverage scale across the genomic window is indicated at the top left corner. (**B**) Genome-wide analysis of reads observed in the 3′ region of snRNAs containing an Sm site sequence (*n* = 30; see Supplementary Table S2) in RNA-seq experiment performed on poly(A)^+^ RNA isolated from control (C702) and AC438/AC439 patients’ cells. Data are presented as dot plots with mean. ns (non-significant), **(*P* < 0.01) represent statistical significance as determined by one-way ANOVA with post-hoc Tukey's test. (**C–F**) Views of the genome browser illustrating reads for the indicated snRNAs in control (C702) and AC438/AC439 patients’ cells. The chromosomal location of each gene is shown and the read coverage scale across the genomic window is indicated at the top left corner of each panel. The size scale corresponds to 100 bp. Means of two replicates are shown for all tracks.

To explore the consequences of the g.108_126del;g.111G>A mutations on gene expression and pre-mRNA splicing on a genome-wide level, we performed deep sequencing on poly(A)^+^ RNA extracted from control and patients’ lymphoblastoid cells. Concerning the *RNU4ATAC* gene, analysis of RNA-seq tracks revealed, as expected, hardly any reads for the control given that mature U4atac snRNAs are not polyadenylated (Figure 4A). Surprisingly, 3′-extended U4atac mutant transcripts can be detected in the AC438 and AC439 patients’ cells (Figure 4A). They correspond to both U4atac mutated species, as a drop in reads covering the 108_126del region can be easily detected in the genome browser view. To analyze whether accumulation of 3′-extended species is specific to U4atac snRNA in the patients’ cells or occurs for other snRNAs, we selected the reads covering the 3′ region of minor and major snRNAs containing an Sm-binding site. By examining the list of 30 spliceosomal snRNA genes extracted from two technical replicates (Supplementary Table S2), we found that an increase in 3′-extended snRNA transcripts is observed in the patients’ cells (Figure 4B). This occurs for the minor U11 and U12 snRNAs (Figure 4C and D, respectively) as well as for major snRNAs, as for example U4 and U5 encoded by *RNU4-2* and *RNU5D-1* (Figure 4E and F, respectively) or snRNA variants encoded by *RNVU1-14* and *RNVU1-4* genes (Supplementary Figure S2A, B). In contrast, no 3′-extended RNA transcripts are observed for *RNU6ATAC* transcribed by RNApol III or for the scaRNA10, a small nucleolar RNA (snoRNA) processed from debranched pre-mRNA introns (Supplementary Figure S2C, D). Altogether, our data show that 3′-extended snRNA species can be detected at higher levels in AC438 and AC439 lymphoblastoid cells compared with the control.

### Characterization of 3′-extended U4atac108_126del and U4atac111G>A RNA species produced in AC438 and AC439 lymphoblastoid cells

**Figure 5.**
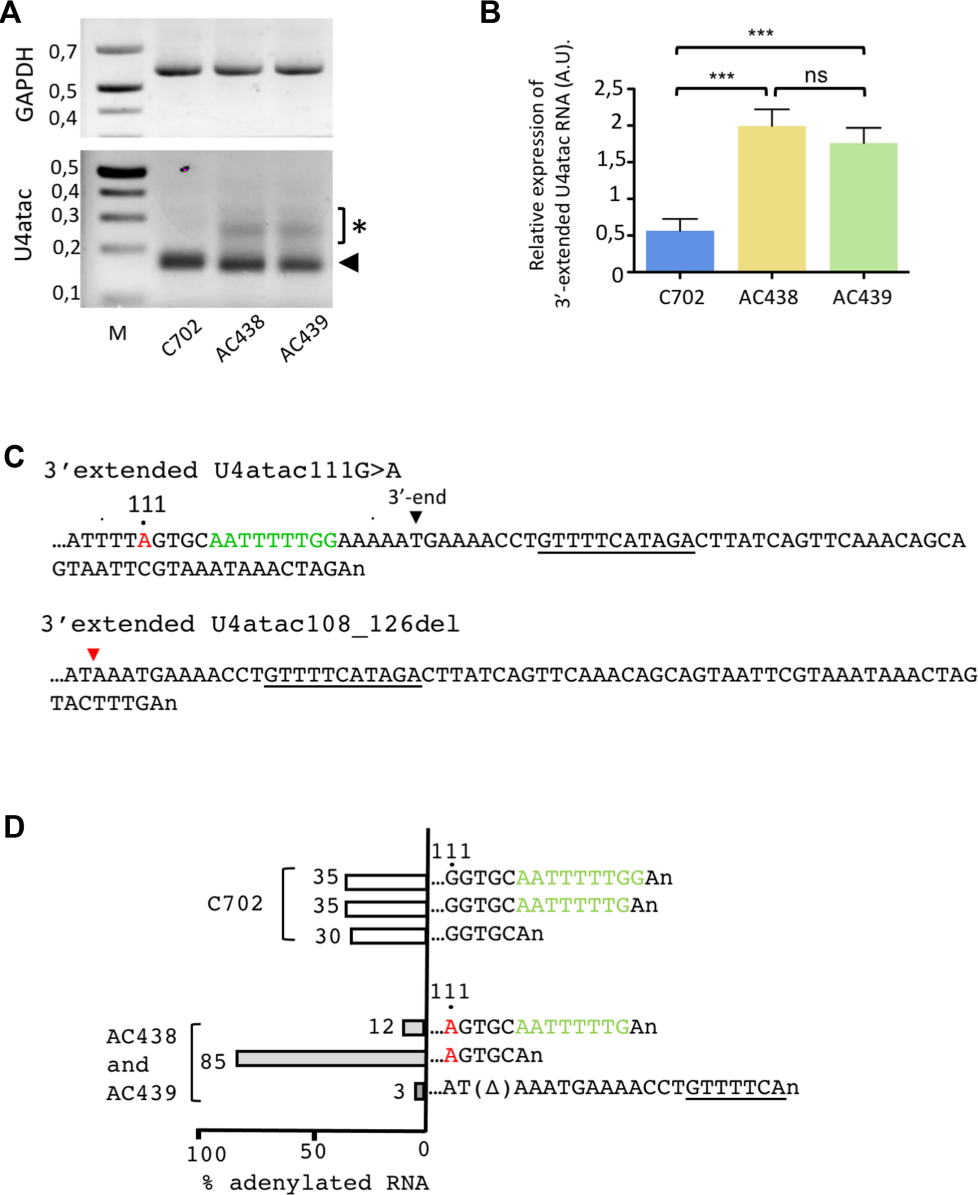
Production of 3′-extended U4atac mutant RNA species. (**A**) RT–PCR analyses of 3′-extended U4atac transcripts and poly(A)^+^ GAPDH transcripts in control and AC438/439 patients’ cells. The asterisk indicates PCR products corresponding to U4atac extended RNA species and the arrowhead indicates adenylated U4atac processing intermediate products. The sizes of the marker (M) are shown on the left and are in kilobases. (**B**) Expression levels of 3′-extended U4atac were analyzed after normalization to GAPDH signals from three trials. Data are presented as bar graphs with the SD. ns (non-significant), ***(P < 0.001) represent statistical significance as determined by one-way ANOVA with Tukey's test. (**C**) The sequences of the U4atac 3′-extended RNA species found in patients’ cells are shown. The 111G>A mutation is shown in red. The Sm site and the 3′ end of the mature U4atac snRNA are also indicated in green and with a black arrowhead, respectively. The sequence showing similarities with the 3′-box element required for snRNA maturation (13) and found in the genomic region downstream of the *RNU4ATAC* gene is underlined. The red arrow indicates the position of the deletion observed in the U4atac108_126del mutant. (**D**) Sequences and analyses of adenylated processing intermediates found in control (C702) and AC438/AC439 patients’ cells. The Sm site is shown in green and the 111G>A mutation in red. Δ indicates the 108_126 deletion. The section of the 3′-box sequence found in the adenylated U4atac108_126del transcript is underlined. The frequency of each product is indicated.

We next confirmed the presence of the 3′-extended U4atac products in AC438 and AC439 cells by selectively amplifying the 3′ ends of cDNAs in 3′ RACE experiments using an oligo(dT) anchor primer (39). As shown in Figure 5A, similar levels of glyceraldehyde phosphate dehydrogenase (GAPDH) transcripts are found in both control and patients’ cells, demonstrating that equivalent amounts of RNA are used in the different samples. In contrast, higher levels of longer PCR products (marked by an asterisk) are found in the mutants while they are barely detected in control lymphoblastoid cells (Figure 5A, B). These extended 3′ RACE products found in AC438 and AC439 cells were further cloned and sequenced. As shown in Figure 5C, we detected RNA species ending 39 nt downstream of the 3′ box sequence (13) necessary for processing of nascent snRNA transcripts (Supplementary Figure S3). Another RNA species corresponds to the U4atac108_126del form carrying a 3′-extended product ending 46 nt downstream of the 3′ box (Figure 5C). It should be noted that our cloning and sequencing analyses were not performed in-depth and it is likely that additional 3′-extended transcripts might be present in the mutant cells.

In addition to these 3′-extended RNAs, which were not found in control cells, our 3′ RACE experiment gave rise to additional and shorter PCR products (marked by an arrowhead in Figure 5A) both in patients’ and in control cells. Cloning and sequencing of these PCR products revealed that they mainly correspond to adenylated U4atac (for control) and U4atac111G>A (for mutants) products ending just before (…..GGTGCAn) or after the Sm site sequence (…..ATTTTTGGAn or …..ATTTTTGAn) while another PCR product carrying the 108_126del sequence was detected only in the patients’ cells (Figure 5D). These products probably represent adenylated U4atac maturation intermediates, and their comparison shows that the proportion of RNA species without the Sm site is three times more important in the patients’ cells than in the control.

It is important to note that the above-described RNA-seq and 3′ RACE experiments use oligo(dT) primers which can induce some biases, as adenylated RNA species produced during snRNA biogenesis (21) could also be primed by the oligo(dT) primer. This hinders quantitative analysis of snRNA maturation and allows only qualitative conclusions. Further deep sequencing experiments of cDNA libraries constructed using adapter-ligated RNAs are required to precisely quantify the amounts of 3′-extended products of the U4atac mutants and the other minor and major snRNAs.

### Retention of U12 introns in the RNU4ATAC compound heterozygous g.108_126del;g.111G>A U4atac cells

We next performed a bioinformatic analysis of the RNA-seq datasets to quantify intron retention (IR) using a dedicated tool, IRFinder (34). For each annotated intron and for each sample, IRFinder computes the PSI (percent spliced in), a metric evaluating the strength of the retention (0%, no retention; 100%, full retention). Principal component analysis (PCA) of the most variable PSI values of U12 and U2 introns revealed correct clustering for duplicates and that the AC438 and AC439 transcriptome datasets were more similar to each other than to the control (Supplementary Figure S4).

**Figure 6.**
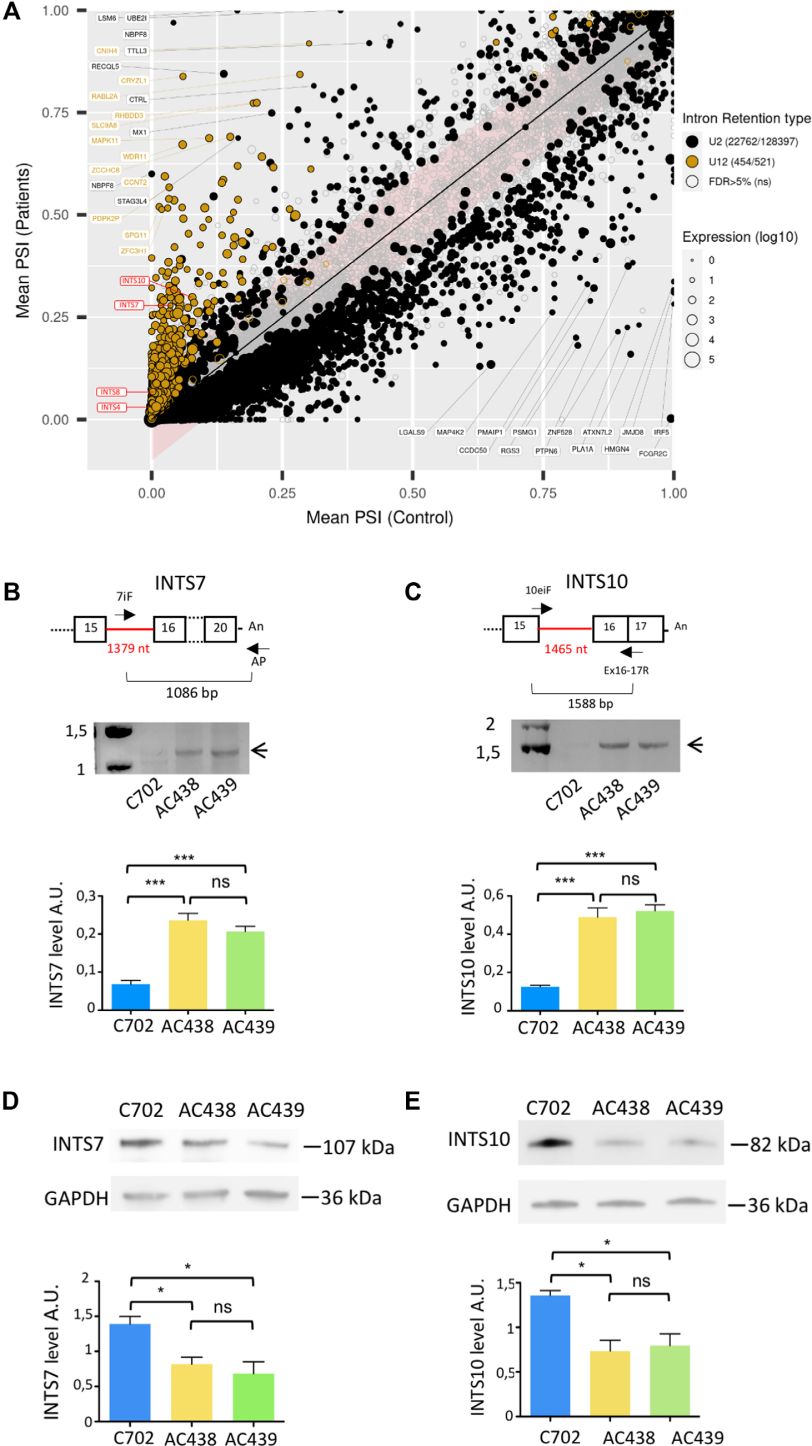
Analysis of splicing defects in compound heterozygous lymphoblastoid cells. (**A**) Plots of the mean U2- and U12-type intron retention levels expressed with the PSI metric and obtained for the patients’ versus the control datasets (PSI-plots). Each circle represents an intron, the color indicates its type and the size indicates the amount of the corresponding transcript. The identity of some introns is shown and the minor introns corresponding to the *INTS4*, *INTS7*, *INTS8* and *INTS10* genes are shown in red. The filling status indicates the significance of the level of intron retention (filled circle, FDR **≤**5%; unfilled circle, FDR > 5%). The intron position with respect to the line indicates whether the intron is more retained in patients (above the line) or control (below the line). (**B** , **C**) RT–PCR analyses of minor introns of *INTS7* (**B**) and *INTS10* (**C**) genes on RNA purified from control (C702) and AC438/439 patients’ cells. The schematic drawing shows the primer used in the experiment and the sizes of the expected fragments if the minor intron is retained, with the corresponding PCR fragment indicated by an arrow. Quantifications are shown below and were performed on three trials. Data are presented as bar graphs with the SD. ns (non-significant), ***(*P* < 0.001) represent statistical significance as determined by one-way ANOVA with Tukey's test. (**D** , **E**) Western blot analysis was performed on extracts from control and AC438/439 patients’ cells using antibodies against INTS7 (**D**) and INTS10 (**E**) proteins. GAPDH served as loading control and was used for quantification (lower panel). Data from three trials are shown and presented as bar graphs with the SD. ns (non-significant), *(*P* < 0.05) represent statistical significance as determined from one-way ANOVA followed by Tukey's test. The molecular weight (kDa) of the proteins is shown on the right.

We then used kissDE (40) to run a differential analysis between MOPD1 patients and the control, and quantify the magnitude of the splicing alteration through ΔPSI (PSI_Patients – PSI_Control). Out of the 251 754 annotated introns, 128 397 U2 and 521 U12 introns were sufficiently covered for the differential analysis, resulting in 22 762 U2 (18%) and 454 U12 (87%) IRs with false discovery rate (FDR) <5% (Supplementary Table S3; Figure 6A). Importantly, 100% of the U12 ΔPSIs were positive, which means that the retention of the intron was higher in the patients versus the control, and 145 (32%) of them were highly affected with ΔPSI >10%. Although IR could lead to mRNA degradation through the nonsense-mediated mRNA decay (NMD) pathway, only 13 U12-containing genes were differentially underexpressed in patients and 19 were overexpressed [DESeq2 analysis with FDR <5% and abs(log2(FC))>2] (Supplementary Table S4). Intriguingly, 19 805 (87%) of the differentially spliced U2 introns had a negative ΔPSI, meaning that the introns were better spliced in the patients compared with the control, a pattern that was observed in a previous study using peripheral blood mononuclear blood cells (PBMC) from Roifman patients but not in MOPD1 fibroblasts and amniocytes (41). The reasons for such a pattern concerning U2 introns remain unknown. Gene Ontology (GO) term enrichment analysis with topGO (42) for U12 introns showing the highest levels of retention revealed terms related to chordate embryonic development, vesicle targeting, non-motile cilium assembly, positive regulation of protein-containing complex assembly, muscle cell differentiation and metal ion transport (Supplementary Table S5). The enrichment of the ‘non-motile cilium assembly’ term is in accordance with our recent report showing links between minor splicing deficiency and cilium dysfunction (43).

### The production and assembly of Integrator subunits are altered in patients' cells

As already mentioned in the Introduction, the 3′-end processing of the spliceosomal snRNAs requires an endonucleolytic cleavage of the nascent snRNA transcripts by the INTS11 RNA endonuclease component of the Integrator complex (11,44–46). Given that the Integrator complex is essential for 3′ end processing of nascent snRNA transcripts, we hypothesized that the presence of 3′-extented snRNAs might be due to alterations in the homeostasis and function of the Integrator complex. In this regard, it is important to note that out of 14 proteins making up the Integrator complex, four subunits (INTS4, INTS7, INTS8 and INTS10) are encoded by genes containing a minor intron. Closer inspection of retained U12 introns (Supplementary Table S3; Figure 6A) showed high ΔPSI values for *INTS7* and *INTS10* (23 and 20, respectively) while ΔPSI values are lower for *INTS4* and *INTS8* (2 and 6, respectively). The distribution of the reads of the Integrator subunits *INTS7* and *INTS10* genes between mutant and control cells is depicted in Supplementary Figure S5, which clearly shows IR, a result in agreement with the high ΔPSI. RT–PCR validation experiments performed on total RNA from mutant and control cells confirm strong IR in *INTS7* and *INTS10* (Figure 6B, C). Moreover, using western blot analyses, we further found significant reduced levels of INTS7 and INTS10 subunits in the patients’ cells (Figure 6D, E). In contrast, the levels of the other Integrator subunits including INTS4 and INTS8 are not significantly changed in the patients when compared with the control (Supplementary Figure S6).

**Figure 7.**
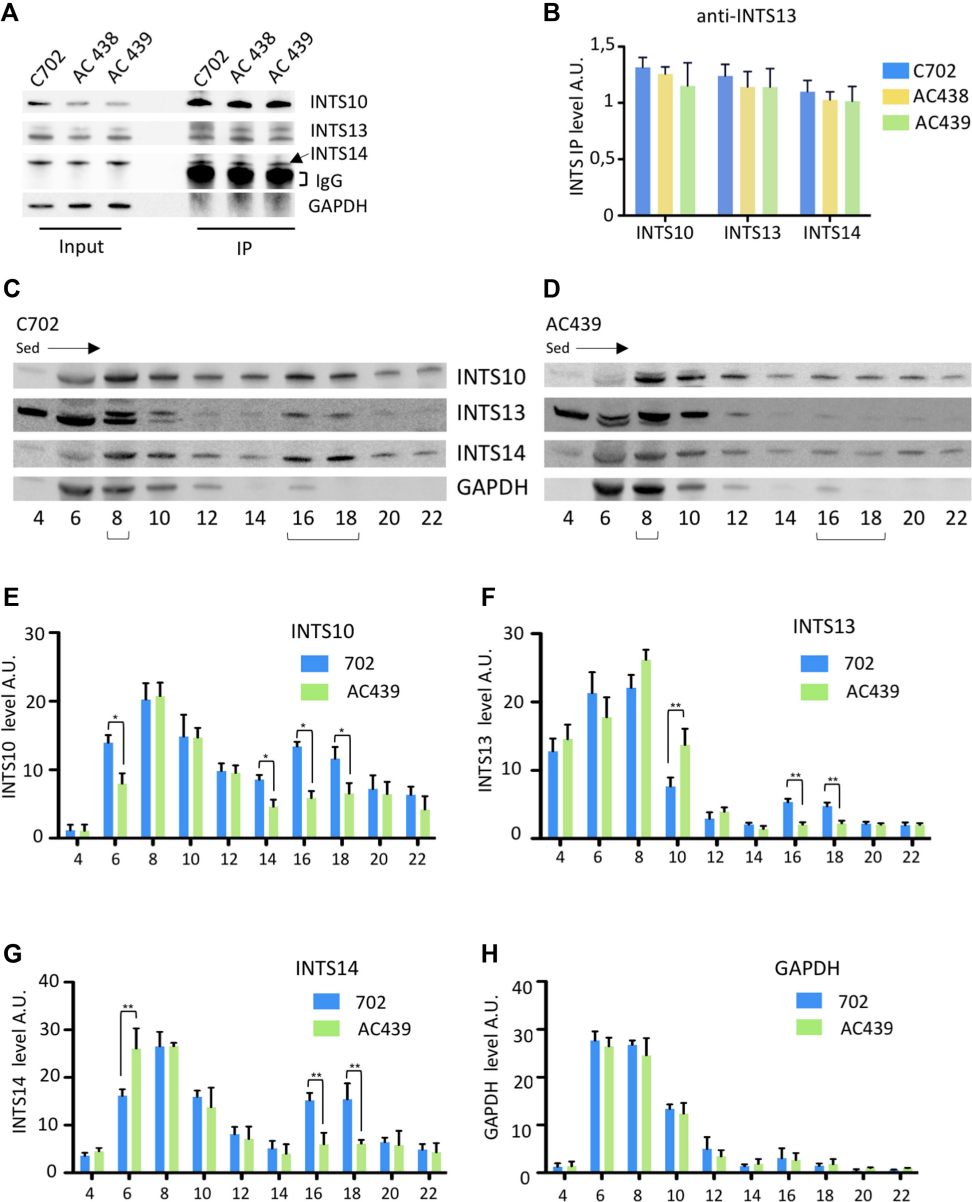
Analyses of the INTS10/13/14 module. (**A**) Extracts from control C702 and AC438/AC439 patients’ lymphoblastoid cells were used in immunoprecipitation experiments with anti-INTS13 antibodies. The input and the immunoprecipitated proteins were analyzed by western blot. (**B**) Quantification of the levels of immunoprecipitated INTS proteins was performed from two trials. A.U., arbitrary units. Error bars represent the SD. No significant differences are found. (**C, D**) Glycerol gradient sedimentation of extracts prepared from the control (C702) and A439 patient's cells. Extracts were fractionated on a glycerol gradient and proteins in each fraction were analyzed by SDS–PAGE and western blot. Numbers of fractions are indicated and the positions of INTS10/13/14 peaks are shown with a bracket. The direction of sedimentation, indicated by the arrow, is from top to bottom. (**E–H**) Quantification of INTS10, INTS13 and INTS14 levels in control and AC439 patient’s cells was performed from two trials by using GAPDH as loading control. A.U., arbitrary units. Data are presented as bar graphs with the SD. *(*P* < 0.05) and **(*P* < 0.01) represent statistical significance as determined by two-way ANOVA.

Various experiments showed that the Integrator complex assembles in a stepwise manner from separate stable modules including the shoulder module (INTS5/8), the backbone module (INTS1/2/7/12), the cleavage module (INTS4/9/11) and the ternary complex INTS10/13/14 which has been shown to bind RNA (47,48). Based on our finding that INTS10 levels decrease in patients’ cells, we first tested whether limited amounts of INTS10/13/14 are immunoprecipitated using anti-INTS13 antibodies. This is not the case since approximately similar levels of INTS10, INTS13 and INTS14 are found in the pellet from control and mutant cells (Figure 7A, B). However, it should be noted that the low titer/affinity of the anti-INTS13 antibodies hinders accurate quantification of the INTS10/13/14 module in control and mutant cells since only a very small amount is bound in such experiments.

We next investigated the sedimentation behavior of the INTS10/13/14 module by performing glycerol gradient fractionation of extracts purified from control and AC439 patient cells followed by western blot analysis. As shown in Figure 7C, a first peak containing the three proteins is found in fraction 8 and a second peak in fractions 16–18 of the gradient using extracts from control cells. Based on the distribution of molecular mass markers on similar glycerol gradients, the first peak probably corresponds to the ternary INTS10/13/14 complex with a mass of 226 kDa and the second peak to particles of ∼1 MDa which represent higher order intermediates of the Integrator complex. Formation of these large macromolecular Integrator complexes appears to be impaired in patients’ cells since only the major peak in fraction 8 is clearly visible while the INTS10/13/14 signals in fractions 16–18 are strongly decreased (Figure 7D). Quantification using GAPDH as control indicates that in patients’ cells, fractions 16–18 only contain trace amounts of INTS13 and 2-fold lower levels of INTS10 and INTS14 subunits (Figure 7E–H). Overall, these studies indicate that the higher order assembly of the Integrator complex could be altered in patients’ cells.

## DISCUSSION

In this report, we used cells derived from *RNU4ATAC* compound heterozygous (g.108_126del;g.111G>A) twins with MOPD1 phenotypes to analyze the molecular effects of the mutations on snRNP formation and minor splicing. Our results show that compound heterozygous lymphoblastoid cells contain lower levels of U4atac snRNA, and, in turn, of minor di- and tri-snRNPs, when compared with control, due to the instability of U4atac108_126del and a slightly lower level of U4atac111G>A. Our work also reveals that patients’ cells contain 3′-extended snRNA transcripts. We show moreover that minor introns are more retained in the mutants, that the amounts of Integrator subunits INTS7 and INTS10 are decreased and that mutant cells display differences in the assembly of Integrator subunits.

### Decreased levels of minor di- and tri-snRNPs in compound heterozygous g.108_126del;g.111G>A patients

Our results show that levels of U4atac snRNA are decreased by ∼55% in the compound heterozygous g.108_126del;g.111G>A cells when compared with the amount of U4atac found in a healthy control carrying two wild-type copies of the *RNU4ATAC* gene. This leads to lower levels of minor di- and tri-snRNPs, which could be responsible for the observed splicing defects. However, the 3′ stem–loop carrying the U4atac111G>A mutation may play an additional role in reducing critical RNA–protein contacts required for the structural rearrangements occurring during spliceosome formation (see discussion below).

Our 3′ RACE and RNA-seq analyses showed also that patients’ cells contain 3′-extended U4atac mutant transcripts as well as other 3′-extended snRNA species that are not found in control cells (Figure 4) and whose proportions cannot be estimated due to potential biases in the preparation of the libraries. The presence of 3′-extended snRNA species can be explained by a dysfunction of the Integrator complex, which could be responsible for the reduced levels of U4atac111G>A to a critical threshold in the patients’ cells. In contrast, the steady-state amounts of other snRNAs including snRNAs from the major spliceosome are not affected (Figure 2B–D), and this could be due to the fact that the amounts of the major snRNAs are 100 times higher compared with the minor snRNAs (8,9).

We were not surprised that the U4atac108_126del mutant form lacking the entire Sm site was undetectable by northern analysis. Indeed, the Sm protein-binding site (consensus: PuAU_3-4_NUGPu) is highly conserved among the snRNAs in eukaryotes and is the primary determinant for snRNP stability (49,50). Accordingly, previous studies showed that the Sm site is required for the production of stable trypanosomatid U5 snRNA (51) and that depletion of Sm proteins in yeast leads to the degradation of all U snRNAs except U6 (52,53). Inability to detect the U4atac108_126del mutant is likely to be due to its increased susceptibility to degradation by nucleases. In this regard, the DEDD family deadenylase TOE1 has been recently identified as being critical for snRNA 3′ trimming in human cells and it has been proposed that TOE1 is at the center of a quality control pathway that segregates regular snRNAs from unstable variants (21,22). It is thus plausible that U4atac108_126del and U4atac111G>A RNA species with aberrant 3′ ends are not protected by TOE1 and thus become substrates for degradation by the nuclear RNA exosome. A competition between TOE1 and the nuclear RNA exosome has indeed been observed in a recent study showing that TOE1 knockdown destabilizes a U12 snRNA mutant while knockdown of MTR4, a component of the nuclear exosome targeting (NEXT) complex, has an opposite effect (54).

### Function of the 3’ stem–loop of U4atac snRNA

As already mentioned above, Integrator is a metazoan-specific complex of at least 14 subunits which interacts tightly with the RNApol II C-terminal domain and is required for the formation of the 3′ end of snRNAs (11,55–57). It is tempting to propose that the apical 3′ stem–loop of U4atac is recognized by the Integrator complex and that mutation 111G>A, as well as mutation 108_126del, impedes such an interaction, precluding efficient transcription termination and co-transcriptional 3′-end cleavage of the nascent snRNA mutant forms. While it will be important to establish if the 3′ stem–loop of U4atac interacts directly with the Integrator and to what extent the mutations hinder such binding, several observations suggest that this is likely to be the case. First, we found that the mutant cells contain 3′-extended U4atac108_126del and U4atac111G>A transcripts. Accordingly, misprocessed and uncleaved forms of snRNAs as well as accumulation of aberrant polyadenylated U1 transcripts is a hallmark of a defective Integrator function in 3′-end processing of snRNAs (58–60). Second, it has also been shown that terminal stem–loops within U2 and U7 snRNAs promote 3′-end processing (61,62). Moreover, it is known that Integrator disruption in *Caenorhabditis elegans* causes transcription of genes located downstream of the snRNA loci, generating long chimeric sn-mRNAs (63). Likewise, depletion of INTS11 is sufficient to induce readthrough transcription at hundreds of genes, giving rise to downstream of gene (DoG) transcripts (64). Recent studies show also that the INTS10–INTS13–INTS14 module binds preferentially to RNA stem–loop structures and stabilizes association of the cleavage module INTS4–INTS9–INTS11 to the target RNAs (47). Finally, an accumulation of polyadenylated major and minor snRNA species has also been observed in RNA-seq experiments performed on poly(A)^+^ RNAs purified from a lymphoblastoid cell line derived from a MOPD1 patient carrying the U4atac51G>A mutation in the 5′ stem–loop (41). This indicates that the function of Integrator in snRNA 3′-end cleavage might also be affected in this mutant which has been shown to be defective in minor tri-snRNPs formation (30).

Our immunoprecipitation experiments showed that the U4atac111G>A mutation does not hinder the binding of the common Sm core proteins to the snRNA (Figure 2E, F). The 111G>A mutation also does not affect the association of the U4atac mutant with U6atac because no free U4atac111G>A RNPs can be detected in the glycerol gradient sedimentation experiments (Figure 3A–C). Accordingly, free U4 and U4atac snRNPs have never been isolated and do not accumulate in human wild-type cellular lysates as free particles but are always found associated with U6 and U6atac snRNAs, respectively (30,65–68). It is noteworthy that a yeast U4-3′Tryp mutant, carrying the shorter 3′ stem–loop of *Trypanosoma brucei*, sediments as a free particle and it has been proposed that one role of the U4 3′-terminal domain might be to prevent the formation of a competing structure in the 5′ region of the U4 snRNA (69). The fact that the U4atac111G>A snRNA is still able to form minor di- and tri-snRNPs suggests that the 111G>A mutation does not alter the overall secondary and tertiary structures of the snRNA. Although the entire deletion of the 3′ stem–loop of human U4atac abolishes *in vivo* splicing (70), a negative effect of the 111G>A mutation on the structure of the U4atac111G>A snRNA and on the formation of di-snRNPs can further be ruled out based on previous mutational analyses on human and yeast U4 snRNAs. Indeed, deletion of the 3′ stem–loop in human nuclear extracts and *Xenopus* oocytes does not significantly inhibit di-snRNP formation and splicing (71–74), and a series of point mutations in the 3′ stem–loop of yeast U4 were found to be functional *in vivo* and *in vitro* (75,76).

While the atomic features of the activated minor spliceosome have been determined (77), no relevant high-resolution structure of a pre-catalytic minor spliceosome with U4atac is available, excluding a detailed analysis of the components surrounding the 3′ stem–loop. However, the structure of a pre-catalytic B-complex of the major spliceosome shows that the 3′ domain of the U4 snRNA is in close contact with Brr2 and other components both in human and in yeast (78–80). If applied to U4atac, it is possible that contacts between proteins and the 3′ stem–loop of U4atac could be required for the proper structural rearrangements occurring during assembly of the minor spliceosome and that the 111G>A mutation could disrupt such critical RNA–protein interactions. The splicing defects observed in the compound heterozygotes could thus be due to a combination of a reduced amount of U4atac111G>A and reduced ability of its 3′ stem–loop for formation of critical RNA–protein interactions necessary for spliceosome formation.

### Alterations in U12 splicing and in Integrator integrity in the compound heterozygous patients’ cells

Our results also demonstrate that splicing of minor introns is affected in the compound heterozygous g.108_126del;g.111G>A *RNU4ATAC* cells, giving rise to U12 intron retention. This is in agreement with previous studies showing that splicing of U12-type introns is severely affected in lymphoblastoid cells from MOPD1 patients as well in models of Roifman and Lowry Wood syndromes carrying *RNU4ATAC* mutations (26,28,41,81,82). Close examination of available datasets indicates that minor introns of *INTS7* and *INTS10* are found with elevated and highly significant retention indexes (41,82), as is the case in our work. Importantly, our results show for the first time that retention of *INTS* minor introns, which was confirmed by RT–PCR experiments (Figure 6B, C), led to a significant reduction in the quantity of INST7 and INTS10 proteins. Moreover, we found also that formation of large macromolecular Integrator complexes is impaired in mutant cells (Figure 7C–H), suggesting that the Integrator complex homeostasis could be affected. This view is consistent with previous studies showing a reduction in integrity and function of the Integrator complex in three individuals carrying a homozygous truncating *INTS1* variant and three siblings harboring compound heterozygous *INTS8* mutations, all presenting with severe neurodevelopmental delay (59). Cells from these patients show reduced amounts of several INTS subunits (including INTS3, INTS5, INTS11 and INTS12), with a significant reduction of INTS4 and INTS9 protein levels. Interestingly, this last study reports moreover that Integrator-deficient patient cells display altered splicing patterns and differential gene expression indicative of global transcriptome perturbations (59). Minor snRNAs were not analyzed in this study, but it was shown that cells from patients with *INTS8* mutations have increased levels of unprocessed U1, U2 and U4 snRNAs while total U snRNA levels did not change. It is also noteworthy that depletion of INTS11 by small interfering RNA (siRNA) gives rise to an increase of 3′-extended RNU11 transcripts in HeLa cells (83). Finally, other studies show that Integrator subunits bind to and control the outputs of many loci in addition to snRNAs, such as for example protein-coding genes, enhancer RNA genes and long non-coding RNA genes (84–89). While the expression of many protein-coding and non-coding RNA genes was altered upon depletion of Integrator subunits, no significant alterations could be observed in the levels of snRNAs, suggesting that changes in the transcriptomic profiles were not caused by extensive splicing defects (88–90). These observations highlight that splicing defects of minor introns as well as alterations in the transcriptomic profile could both contribute to MOPD1, Roifman and Lowry Wood diseases.

## DATA AVAILABILITY

RNA-seq data have been deposited in NCBI’s Gene Expression Omnibus (GEO) under accession GSE197344. The data are available at: https://www.ncbi.nlm.nih.gov/geo/query/acc.cgi?acc=GSE197344.

## Supplementary Material

gkac1182_Supplemental_Files
