# Supplementary material for: Mutations in the non-coding RNU4ATAC gene affect the homeostasis and function of the Integrator complex
Source: Nucleic Acids Res. 2022 Dec 20;51(2):712–27. doi: 10.1093/nar/gkac1182 (PMC9881141; doi:10.1093/nar/gkac1182)
Supplement: gkac1182_Supplemental_Files [file gkac1182_supplemental_files.zip › Supplement FigRev2 final.pdf]

Supplementary Information

Mutations in the noncoding *RNU4ATAC* gene affect the homeostasis and function of the Integrator complex

Fatimat Almentina Ramos Shidi, Audric Cologne, Marion Delous, Alicia Besson, Audrey Putoux, Anne-Louise Leutenegger, Vincent Lacroix, Patrick Edery, Sylvie Mazoyer and Rémy Bordonné*

*corresponding author: remy.bordonne@igmm.cnrs.fr

Supplementary Figure S1

Supplementary Figure S2

Supplementary Figure S3

Supplementary Figure S4

Supplementary Figure S5

Supplementary Figure S6

Supplementary Table S1 (separate file)

Supplementary Table S2 (separate file)

Supplementary Table S3 (separate file)

Supplementary Table S4 (separate file)

Supplementary Table S5 (separate file)

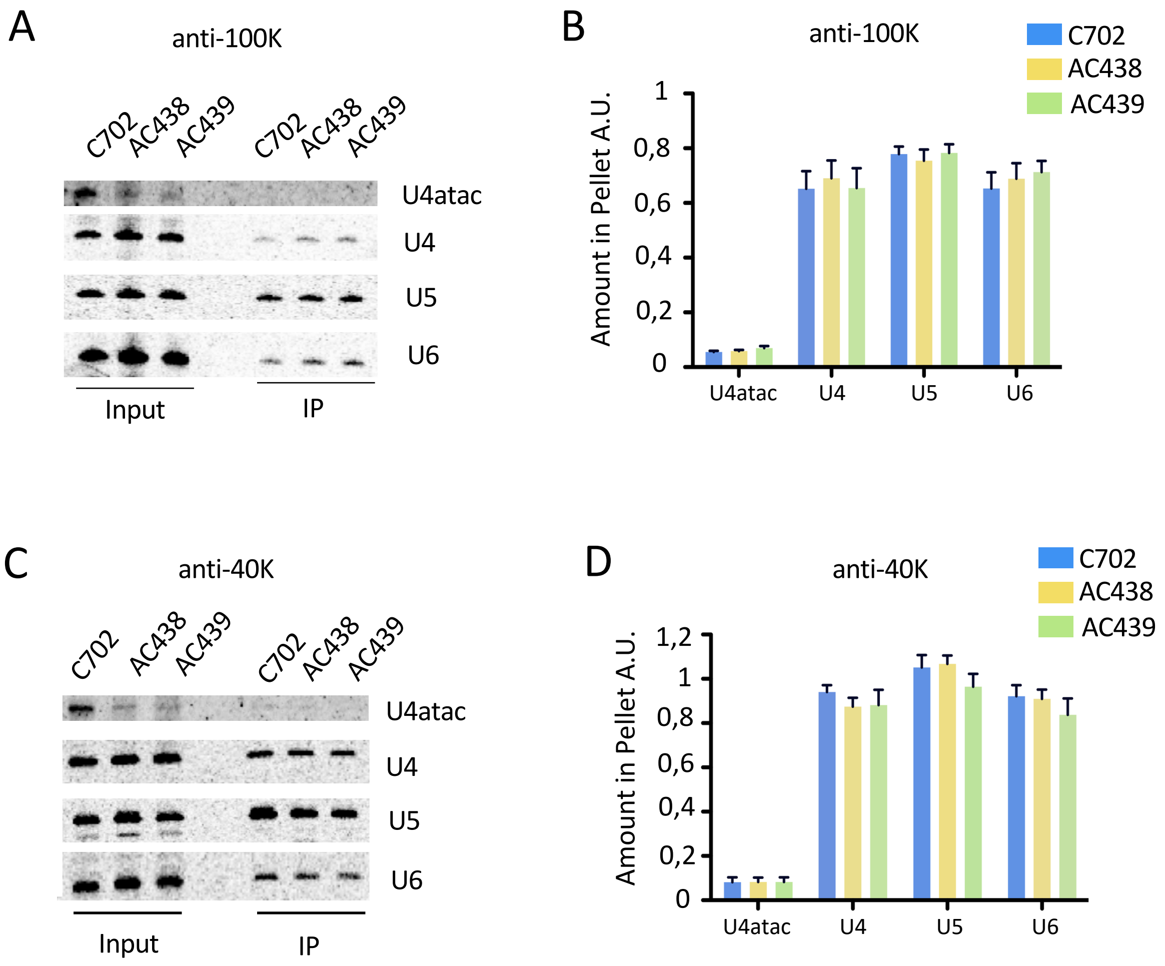

Supplementary Figure S1: Immunoprecipitation experiments. Extracts from control C702 and AC438/AC439 patients' lymphoblastoid cells were used in immunoprecipitation experiments with anti-100K (A) and anti-40K (C) antibodies. The input and the immunoprecipitated RNAs (IP) were analyzed by Northern blot and probed with radiolabeled oligonucleotides complementary to the indicated snRNAs. Quantification (B, D) of the amount of snRNAs found in the pellet was performed from two trials; A.U.: arbitrary units. Error bars represent standard deviation. No significant differences in immunoprecipitated snRNA levels are found in mutant compared to control cells.

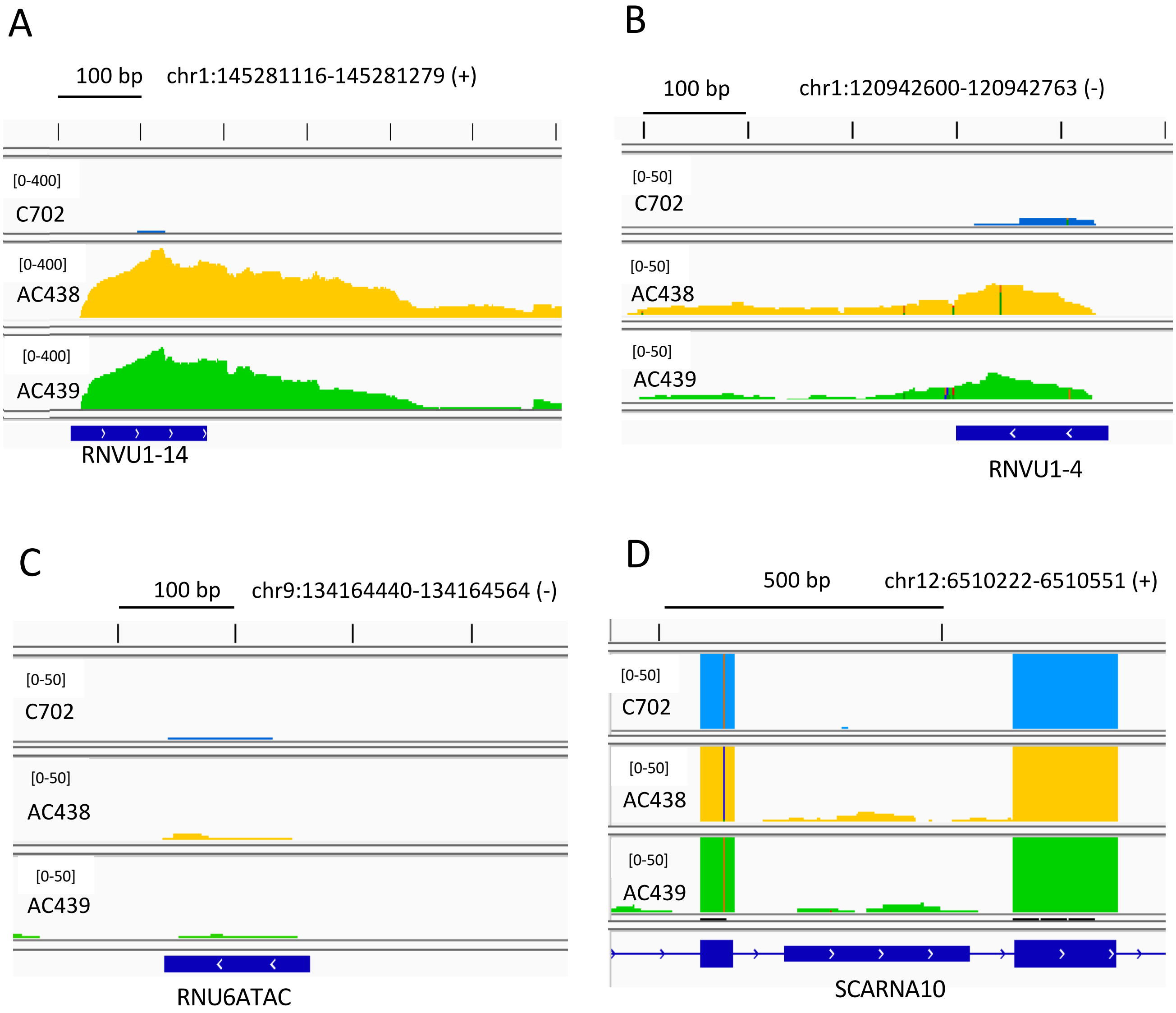

Supplementary Figure S2: (A-D) Genome browser views illustrating reads for the indicated RNAs in control and AC438/AC439 mutant cells obtained in the RNA-seq experiments performed on poly(A)$^+$ RNAs. The chromosomal location of each gene is shown and the read coverage scale across the genomic window is indicated at the top left corner of each panel. Means of two replicates are shown for all tracks. The size scale is also shown. The *RNU6ATAC* and *SCARNA10* genes are shown as negative controls (see text for details).

	3' box
RNU5E-1	GCTTATTTTAAGTGTTTTAAAAACAGATGCGATTCCG
RNVU1-15	CTGGTGCTTCTGTGGTGCGAATAGTAGGTGAGCCGTA
RNVU1-2A	CTGATTTCTTTTTTTCTGGTTTCAAAAATAGACCGTA
RNVU1-14	CTTGGTGCTTCTGTGGTGCGAATAGTAGGTGAGCCGT
RNVU1-31	CTGAATTTTTGTAATGAAAAAATAGACGGCAAGGGTT
RNVU1-6	CTGTATTTTTGTCGTTTTAAAAATAGGTTTTGTTTGC
RNVU1-25	TCTGGAGGTTGTAGTGCAAAAAGCAGTTTGTGTACCA
RNVU1-7	CTGAATTTTTGTAATGAAAAAATAGACTCCCCTATAA
RNVU1-2	CTGATTTTTTGTAGTTTCAAGAATACGCTGTACTCTA
RNVU1-3	CCTGGATTGCTGTCGTTTTAAAAGTGGATTTTGTTTG
RNVU1-27	CTGGCGCTTTTGTGGTGCGAATAGTAGATGAGCCGTA
RNVU1-30	CTGGGTTGTTGTCGTTCTAAAAGTGGATTTTGTTTGC
RNU5A-8P	CAGATCATGTTTTATAAAAAAAGACTTAAAGAGGAAA
RNU5F-1	GCTGAATGTTCTGTTACTAAAGAGAGACGTGTGGGTG
RNU5D-1	GTTCATCAACGGTGTTTAAAAATCAGATAGAAAGTTG
RNU5E-6P	GCTACATTTTTAAGTATTAAAAGTTAGGCAACTACAA
RNU4-2	AGACTGAATTTTTAAAAGTCTAAAGAAAAGGGTTTTA
RNU4-1	AGACTGAATTTTCTTGCAGTTGAACAACAGAGGCTTT
RNU5B-1	GCTATATAAAGCTGTTAAAAAATCAGATTGACTTCAT
RNVU1-34	CCTGATCTGGCTTGTTTATAAAAGGCGTACGTTGTAT
RNU4ATAC	AAAATGAAAACCTGTTTTCATAGACTTATCAGTTCAA
RNU2-63P	GCTAACCATTAGTGACAAAAACAGGTTTGTAATTTTG
RNU12	CCGACCTTATTCACGCCTAAAAAGTAGACTGACTGTG

3' box consensus : $GTttN_{0\text{-}3}AAaADN_{1\text{-}2}AGR$

(where upper- and lowercase letters indicate strict and less strict homology to motif, N is any nucleotide, D=A,G or T, R=A or G)

Supplementary Figure S3: The putative 3'box sequences found in the 3' region of snRNAs and potentially involved in snRNA 3'-end maturation are highlighted in green (13). The 3'-ends of the mature snRNAs are shaded in red. The corresponding 3' box consensus sequence is shown. Adapted from Lykke-Andersen et al. (83).

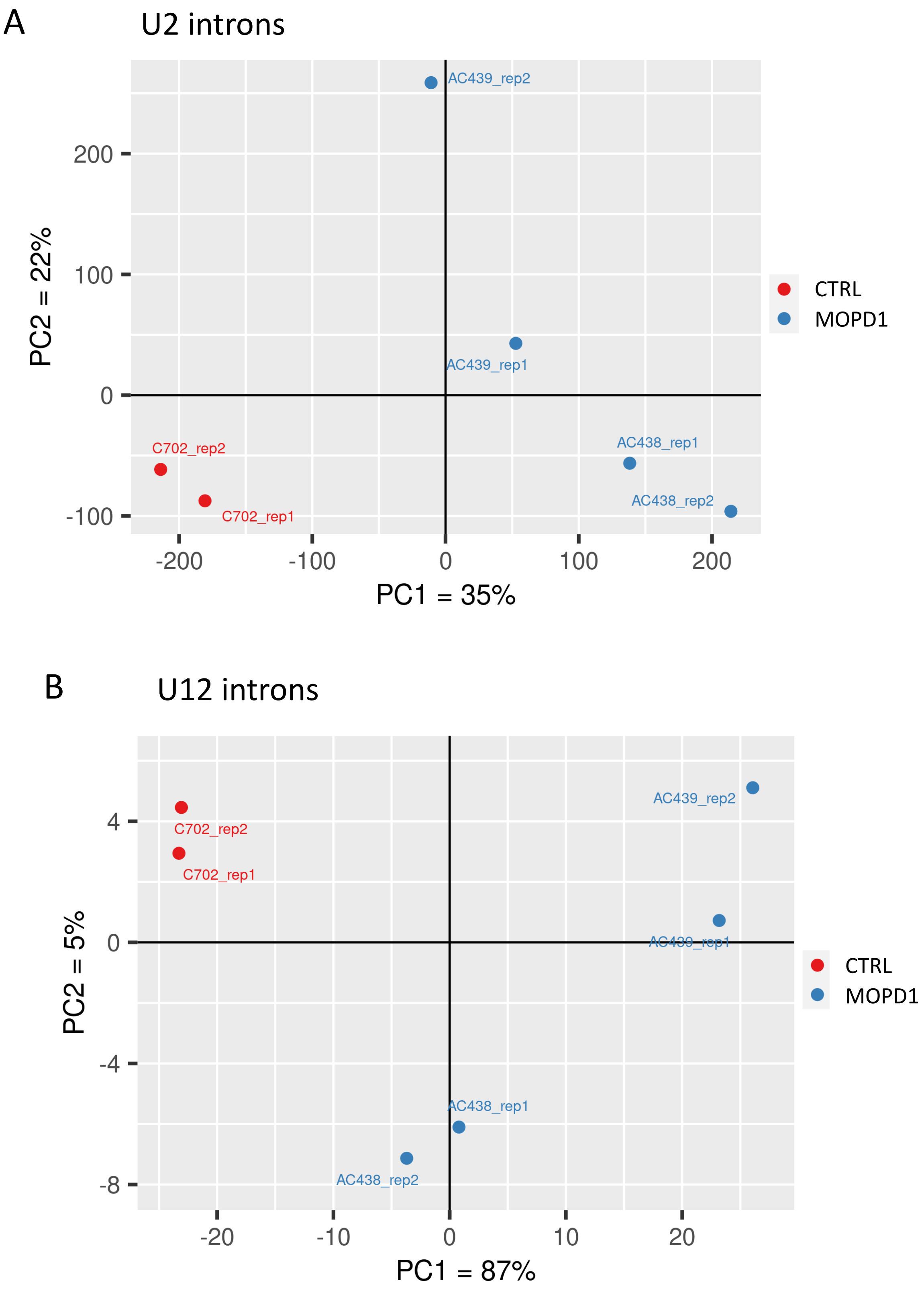

Supplementary Figure S4: Patterns of U2- (A) and U12-type (B) intron retention in C702 control (CTRL) and compound heterozygous AC438/AC439 mutant (MOPD1) cells. Principal component analyses of the most variable mean PSI values of U2 and U12 introns are presented. The analysis was performed on two replicates from control and two replicates from each patient.

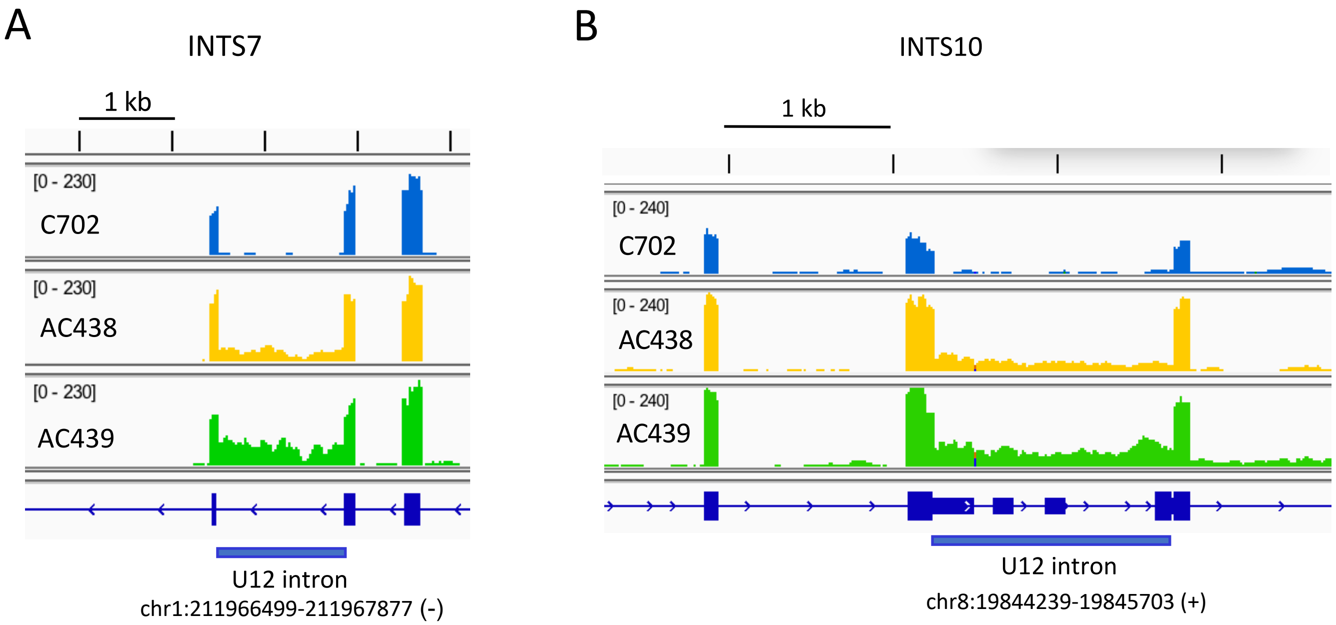

Supplementary Figure S5: Genome browser views illustrating RNA-seq data of *INTS7* (A) and *INTS10* (B) genes containing U12 introns for control C702 and AC438/439 mutant cells. The genomic locations of the corresponding U12 introns are shown below. The read coverage scale across the genomic window is indicated at the top left corner of each panel. Means of two replicates are shown for all tracks. The size scale is shown in kilobase.

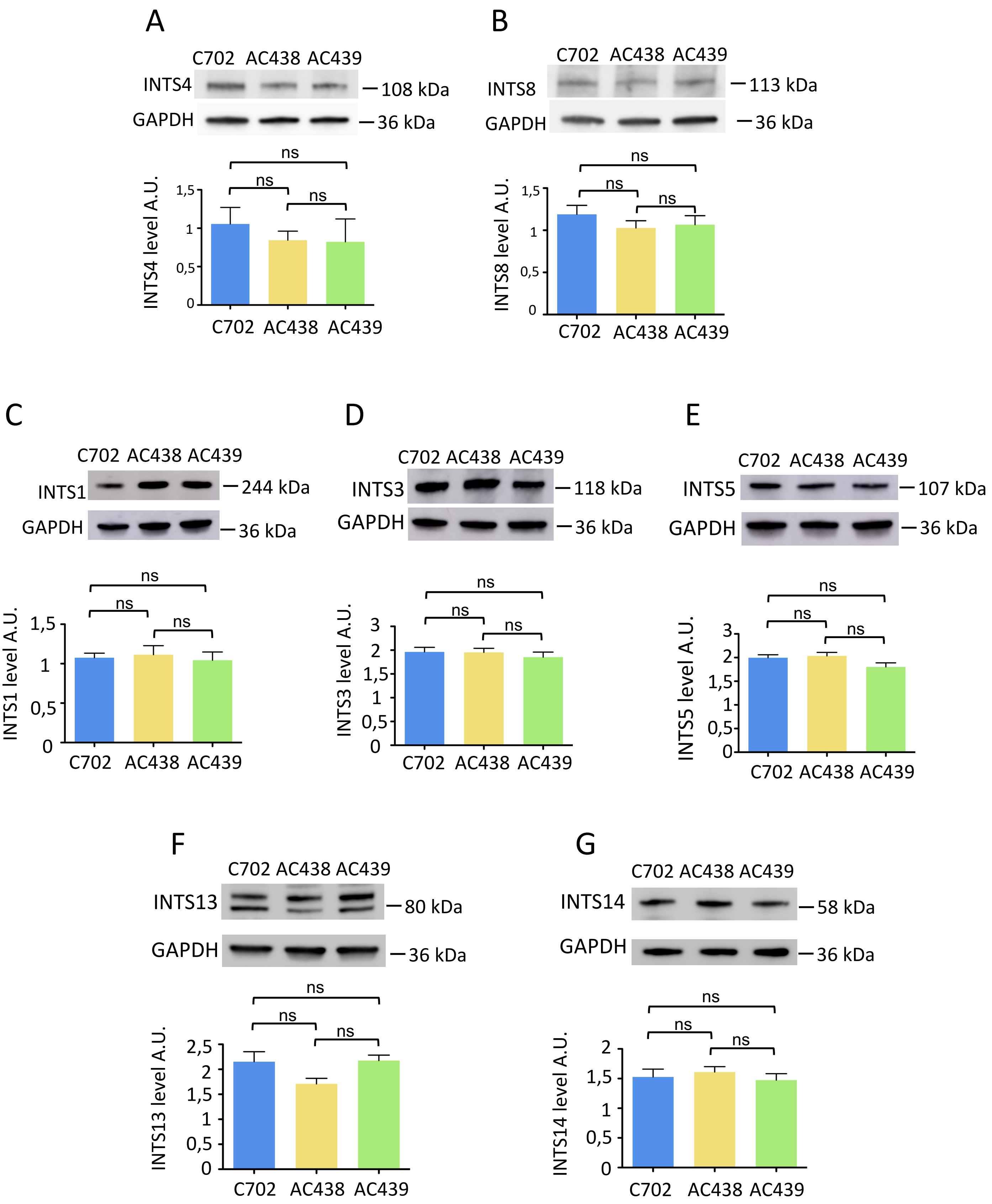

Supplementary Figure S6: (A-G) Western blot analysis was performed on extracts from control and mutant cells using antibodies against the INTS subunits as indicated. Quantification was from three trials and was performed using GAPDH levels as loading control. Data are presented as bar graphs with standard deviation. No statistically significant (ns) differences were found from one-way ANOVA followed by Tukey's test. The molecular weight (kDa) of the proteins is shown at right.

Supplementary Table S1: Lists of oligonucleotides and antibodies used in this study
see file Supplementary Table S1 Oligos and Antibodies.xlsx

Supplementary Table S2: Genome wide analysis of reads observed in the 3’ regions of snRNAs containing a Sm site sequence
see file Supplementary Table S2 Coverage 3’ region Sm snRNA.xlsx

Supplementary Table S3: Lists of genes with retained U2 and U12 type introns
see file Supplementary Table S3 Intron retention U2 and U12.xlsx

Supplementary Table S4: Lists of genes differentially expressed in mutant AC438/AC439 compared to control
see file Supplementary Table S4 Differential Expression.xlsx

Supplementary Table S5: Results of TopGo analyses
see file Supplementary Table S5 TopGo analyses.xlsx
